# Co-Administration of BNT162b2 COVID-19 and Influenza Vaccines in Adults: A Global Systematic Review

**DOI:** 10.3390/vaccines13040381

**Published:** 2025-04-02

**Authors:** Constantina Boikos, Kassandra Schaible, Solange Nunez-Gonzalez, Verna Welch, Tianyan Hu, Moe Hein Kyaw, Laura Elizabeth Choi, Joanna Kamar, Henry Goebe, John McLaughlin

**Affiliations:** 1 Pfizer Inc., Kirkland, QC H9J 2M5, Canada; 2 Evidera, Thermo Fisher Scientific, Waltham, MA 02451, USA; kassandra.schaible@thermofisher.com (K.S.); solange.gonzalez@thermofisher.com (S.N.-G.); joanna.kamar@thermofisher.com (J.K.); 3 Pfizer Inc., New York, NY 10001, USA; verna.welch@pfizer.com (V.W.); tianyan.hu@pfizer.com (T.H.); moe.kyaw@pfizer.com (M.H.K.); 4 Pfizer Inc., Collegeville, PA 19426, USA; laura.choi@pfizer.com (L.E.C.); john.mclaughlin@pfizer.com (J.M.); 5 Pfizer Inc., Tadworth KT20 7NS, UK; henry.goebe@pfizer.com

**Keywords:** BNT162b2, influenza vaccination, COVID-19 vaccine, co-administration, immunogenicity, vaccine safety efficacy, effectiveness, public health, vaccination strategies

## Abstract

Background/Objectives: Co-administration of BNT162b2 with licensed seasonal influenza vaccines (SIVs) is recommended by health authorities. We provide a comprehensive summary of the data supporting this practice in adults. Methods: This systematic review consolidates available evidence on the prevalence, safety, immunogenicity, efficacy, and effectiveness of co-administering BNT162b2 and SIVs. Searches were conducted for English studies in adults ≥ 18 years of age between January 2021 and August 2024, with no geographic restriction. Study quality was assessed using Cochrane RoB 2.0 and the Newcastle–Ottawa Scale. Results: Twenty studies (15 observational and 5 clinical trials) were included, mainly conducted in seven countries in Europe and North America. Eight observational studies reported prevalence, twelve reported safety/reactogenicity, six reported immunogenicity, and three evaluated efficacy/effectiveness. Reported co-administration of BNT162b2 vaccines with SIVs increased over time. Of persons receiving BNT162b2, the proportion that reported co-administered SIVs increased from 2.7% in 2021 to 34.1% in 2023. Although variability in outcomes was observed, no consistent pattern indicating a negative impact on immunogenicity from same-day co-administration was identified. Effectiveness was not observed to change when BNT162B2 was co-administered with SIVs. The incidence of systemic and local adverse events was comparable between individuals receiving the vaccines separately and those receiving them co-administered. Conclusions: The findings from this review indicate that the co-administration of BNT162B2 with SIVs is both safe and effective. This highlights the value of co-administration, which could enhance vaccine uptake by streamlining immunization protocols and reducing health visits.

## 1. Introduction

The administration of multiple vaccines in one medical visit (herein referred to as “co-administration”) is a well-established practice and is recognized as an effective and economical approach to increase immunization rates in pediatric and travel medicine settings [1]. This practice, however, remains underutilized in adults, where suboptimal immunization rates are frequently associated with missed opportunities to vaccinate [2,3]. Health professionals are encouraged to implement co-administration to enhance vaccine coverage and streamline healthcare delivery, especially for seasonal vaccines [1,4].

Given the ongoing burden of respiratory pathogens, particularly influenza, and COVID-19, there is an increasing focus on optimizing adult vaccination strategies [5]. Influenza alone continues to affect up to 1 billion individuals globally in an average year [6,7], with respiratory-related fatalities estimated at 290,000 to 650,000 annually [6,8]. Meanwhile, the World Health Organization (WHO) has documented over 775 million confirmed cases of COVID-19 [9] and more than 7 million COVID-19-related deaths globally [10] between the start of the pandemic in 2020 and May 2024. Although COVID-19 continues to circulate globally, COVID-19 vaccination has saved upwards of 20 million lives worldwide [11], underscoring the critical role of widespread immunization. As a result, health authorities in multiple countries now recommend the co-administration of COVID-19 vaccines with influenza vaccines (IV), among others, to enhance vaccine coverage in adults and provide concurrent protection against these high-burden respiratory diseases [12,13].

Despite these recommendations, the scientific literature has yet to fully summarize the clinical and real-world evidence on the co-administration of the COVID-19 vaccine BNT162b2 with seasonal influenza vaccines (SIVs) in adults. Existing reviews are either not systematic [1] or provide some insights but are not comprehensive in that they (a) only include literature published before 25 April 2022, (b) exclude real-world evidence studies, and (c) do not include data on the prevalence of co-administration [11]. The aim of this systematic literature review (SLR) was to identify and synthesize the evidence regarding the prevalence and outcomes of co-administration, specifically the available evidence on the immunogenicity, efficacy, effectiveness, and safety (including reactogenicity) of co-administration—defined as the administration of the BNT162b2 and SIVs on the same day or during the same medical/pharmacy visit—in both clinical and real-world settings in adults 18 years of age and older. Results from this SLR can inform vaccination strategies by providing evidence on the safety and immunogenicity of co-administering COVID-19 vaccines and IV, potentially supporting policies aimed at optimizing immunization programs.

## 2. Materials and Methods

The SLR was conducted in accordance with the Cochrane Handbook for Systematic Reviews of Interventions [14] and the Preferred Reporting Items for Systematic Reviews and Meta-Analyses guidelines [15] with regard to the methods used to search, identify, review, and summarize the available evidence.

### 2.1. Search Methods and Data Sources

Systematic searches were conducted on 7 August 2024 via OvidSP in MEDLINE, Embase, Cochrane Central Register of Controlled Trials (CENTRAL), and Cochrane Database of Systematic Reviews using a combination of medical subject heading and free-text terms to identify relevant randomized controlled trials (RCTs) and real-world evidence (RWE) studies published since 1 January 2021. Database searches were supplemented by a review of abstracts from relevant scientific conferences (2021–2024), including the European Society of Clinical Microbiology and Infectious Diseases (ESCMID) Global (formerly known as ECCMID), Options for the Control of Influenza organized by the International Society for Influenza and other Respiratory Virus Diseases (ISIRV) and European Scientific Working Group on Influenza (ESWI).

### 2.2. Screening

Eligibility for inclusion was determined using prespecified populations, interventions, comparisons, outcomes, and study design (PICOS) inclusion/exclusion criteria (Table 1). All abstracts and subsequent full texts were screened against prespecified eligibility criteria by two independent investigators, and disagreements were resolved through discussion and consensus or by a third investigator. Studies included in this systematic review focused on study populations 18 years of age and older, including high-risk groups for influenza and COVID-19, who received both COVID-19 vaccines and IV (defined as co-administration). Subgroups of interest were categorized by age: 18–49, 50–64, and 65 years and older. Studies involving individuals under 18 or those without reported COVID-19 or influenza vaccinations were excluded.

The interventions focused on the co-administration of BNT162b2 (in various formulations) and any type of IV, defined as being administered during the same medical visit or within 3 months of each other (for prevalence studies only). Studies involving COVID-19 vaccines other than BNT162b2 or those without SIV co-administration were excluded.

Comparators included any other approved vaccines administered alone, no vaccination, or no vaccination during that season. Outcomes assessed included the prevalence of co-administration, efficacy/effectiveness of the vaccines, immunogenicity, and safety/reactogenicity. Publications that did not report at least one of these outcomes were excluded.

Included studies were clinical trials (phases 1–3), post-hoc analyses, pooled analyses, and observational studies. Excluded were SLRs, pharmacodynamic/pharmacokinetic studies, genetic studies, trials without results, case reports, narrative reviews, and editorials. Only full-text publications, conference abstracts, and letters to the editor published from 2021 onwards in English were considered, with no geographic limits. Preprints older than 6 months were excluded.

All relevant data from the accepted studies were extracted into a prespecified data extraction form by a single investigator and validated by a second investigator. Data were extracted as reported in the studies, with no digitization or calculations. The quality assessment of the included RCTs was conducted using the Cochrane Risk-of-Bias (RoB) assessment tool 2.0. The risk of bias in each of the five domains was classified into three categories (low, some concerns, high), and an overall score was assigned following the algorithm guidance of the RoB 2.0 tool [16]. The quality assessment of the included observational studies and non-randomized trials was conducted using the Newcastle–Ottawa Scale [17].

## 3. Results

**Figure 1:**
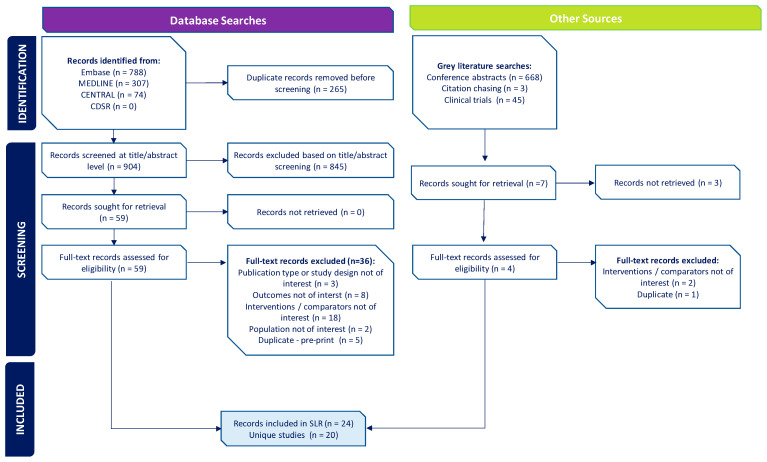
PRISMA Diagram. Abbreviations: CDSR = Cochrane Database of Systematic Reviews; CENTRAL = Cochrane Central Register of Controlled Trials; PRISMA = Preferred Reporting Items for Systematic Reviews and Meta-Analyses; SLR = systematic literature review.

A total of 1169 records were initially identified through database searches, of which 904 unique references were screened at the abstract level after the removal of duplicates. Fifty-nine were retrieved for assessment at the full-text level, twenty-three of which were included in the SLR. One additional publication was identified from the gray literature searches, bringing the final total to 24 publications included in the SLR, reporting on 20 unique studies (Figure 1).

### 3.1. Study Characteristics

Of the 20 included studies, most were observational in design (n = 15, 75.0%) [18,19,20,21,22,23,24,25,26,27,28,29,30,31,32], with 4 randomized clinical trials (20.0%) [33,34,35,36] and 1 non-randomized trial (5.0%) [37]. Most of the studies were conducted in Europe (n = 9, 45.0%) and North America (n = 6, 30%), with 10% conducted in Asia (n = 2), 10% in Oceania (n = 2), and 5% multinational (n = 1). Study sample sizes ranged from 64 [19] to 9,040,176 subjects [25], with follow-up durations ranging from 2 [26] to 280 days [29].

**Figure 2:**
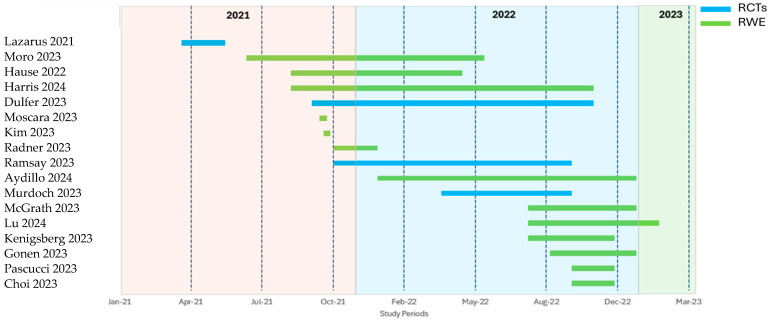
Study periods in the included studies. Abbreviations: RCT = randomized controlled trial; RWE = real-world evidence [18,20,21,22,23,24,25,27,28,29,30,31,33,34,35,36,37].

Eight observational studies reported prevalence [21,22,23,24,25,27,28,30], twelve reported safety/reactogenicity [19,20,22,26,29,31,32,33,34,35], six reported immunogenicity [18,20,31,32,33,34], and three evaluated efficacy/effectiveness [27,29,37]. Randomized clinical trials focused on safety/reactogenicity (n = 4) [33,34,35,36] and immunogenicity (n = 3) [33,34,35]. The single non-randomized trial [37] reported on both safety/reactogenicity, efficacy/effectiveness, and immunogenicity (Appendix A Table A1). The study period was detailed in 85.0% of the studies (n = 17), ranging from April 2021 [34] to February 2023 [25] (Figure 2).

Four RCTs [33,34,35,36] were assessed using the Cochrane RoB tool. Of these studies, two [34,35] were identified as having a low risk of bias, and two [33,36] were assessed as having some concerns, namely related to deviations from intended intervention domains and measurement of the outcome (Appendix A Table A2). As the Cochrane RoB tool is designed only for RCTs, the non-randomized clinical trial and remaining observational studies [18,19,20,21,22,23,24,25,26,27,28,29,30,31,32,37] were assessed using the Newcastle–Ottawa Scale. In the Selection domain, 81% of studies scored 3 points, whereas in the Comparability domain, 88% of studies scored 0 points. Furthermore, in the Outcome domain, 94% of the studies scored 3 points. Overall, the total scores ranged from 6 to 7 points, indicating good quality across the studies (Appendix A Table A3).

### 3.2. Prevalence of Co-Administration

**Figure 3:**
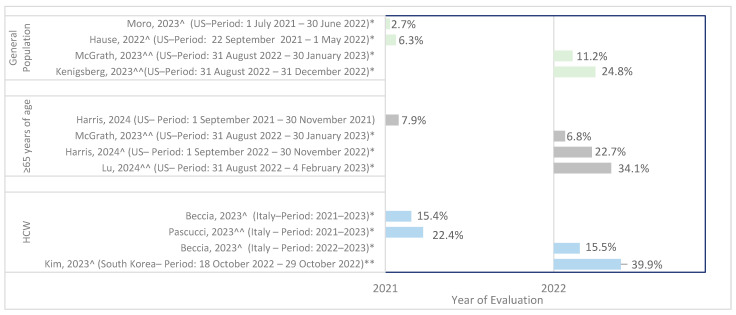
Prevalence of co-administration in RWE. Abbreviations: HCW = healthcare workers, US = United States; ^ original monovalent; ^^ bivalent BA.5; * same day/same visit; ** BNT162b2 booster was subsequently administered 1 week later [21,22,23,24,25,27,28,30,38].

Eight unique studies, reported across ten publications, reported data on the prevalence of co-administration [21,22,23,24,25,27,28,30,38,39], including 67% from the US (n = 6) [21,22,23,24,25,27], 22% from Italy (n = 2) [30,38], and 11% from South Korea (n = 1) [24]. One study was reported in two publications [30,38]. The study periods ranged from July 2021 [28] to February 2023 [25]. In total, the majority of included studies reported co-administration on the same day or during the same visit (n = 8, 89%); only one study reported that the BNT162b2 booster (“booster” refers to any BNT162b2 vaccination other than the primary two-dose series) was administered 1 week after seasonal influenza vaccine (SIV) administration [24]. Five studies focused on the BNT162b2 monovalent booster [21,22,24,28,38], whereas four studies reported on the BNT162b2 bivalent BA.4/5 booster [23,25,27,30]. Eight of these nine studies reported details on the IV, including 33% for recombinant vaccines, adjuvanted vaccines, standard-dose vaccines, and cell-based vaccines, and 22% for live-attenuated vaccines and high-dose vaccines. Regarding valence, eight of the studies included quadrivalent vaccines [21,22,23,24,25,27,28,30], and one study included trivalent vaccines [21] (Figure 3, Appendix A Table A1).

Among the general population, the proportion of persons receiving BNT162b2 booster who also reported receiving a co-administered SIV from 2021 to 2023 ranged from 2.7% between 1 July 2021 and 30 June 2022 [28] to 24.8% between 31 August 2022 and 31 December 2022 [23], calculated among individuals receiving COVID-19 boosters. One study reported co-administration of an SIV and BNT162B2 with a third vaccine (either an adjuvanted vaccine against herpes zoster, a conjugate pneumococcal vaccine, or a polysaccharide pneumococcal vaccine) [27].

Among adults aged 65 years or older, the proportion receiving co-administered BNT162b2 and SIV ranged from approximately 6.8% [21] to 34% (between 31 August 2022 and January/February 2023, respectively) [25]. One study reported the co-administration of an SIV and BNT162B2 with a pneumococcal vaccine [25]. In the studies on older adults, SIV received included high- or standard-dose SIV (not further specified) [26], high-dose adjuvanted quadrivalent Fluzone or Fluad [30,40], and quadrivalent vaccines (the list includes Flublok, Fluzone, Fluad, Flucelvax, or FluMist) [32]. It should be noted that the live-attenuated IV is currently indicated for individuals aged 2–49 years.

Additionally, three studies reported data on the prevalence of co-administration of the BNT162b2 booster with SIV, specifically in healthcare workers [24,30,38,41]. From 2021 to 2023, 15.4% (between 2021 and 2023 [38]) to 39.9% (between 18 October 2022 and 29 October 2022) of healthcare workers in the included studies received the two vaccines at the same time [24]. The frequency of co-administration tended to increase over time in the general population and adults aged 65 years or older. One study conducted in Italy among healthcare workers reported on factors influencing co-administration. All personnel were offered vaccination by the hospital’s hygiene unit from 6 October 2022 to 22 December 2022. Co-administration of both the influenza and anti-SARS-CoV-2 vaccines was encouraged to increase compliance among healthcare workers [30]. No studies reported the prevalence of co-administration in any other specific risk groups or other special populations.

### 3.3. Immunogenicity of Same-Day Co-Administration

#### 3.3.1. Seroconversion and Seroprotection

Seroconversion rates of anti-S immunoglobulin (Ig)G 21 or 28 days after vaccination were reported in three studies [33,34,37]. None of the studies reported seroprotection against SARS-CoV-2.

In one study, the same-day co-administration of BNT162b2 with IIV4 resulted in higher anti-S seroconversion rates in healthy adults compared to sequential administration. At 21 days, co-administration of the BNT162b2 second dose with quadrivalent recombinant influenza vaccine (QIVr) showed an anti-S IgG seroconversion rate of 72%, compared with 61% for BNT162b2 second dose followed by QIVr 21 days later [34]. Similar results were observed with the BNT162b2 monovalent booster and IIV4 at 21 days (83.8% vs. 73%) [33] and with the BNT162b2 bivalent BA.4/5 booster and IIV4 at 28 days (24.7% vs. 16.9%) [37].

**Figure A1:**
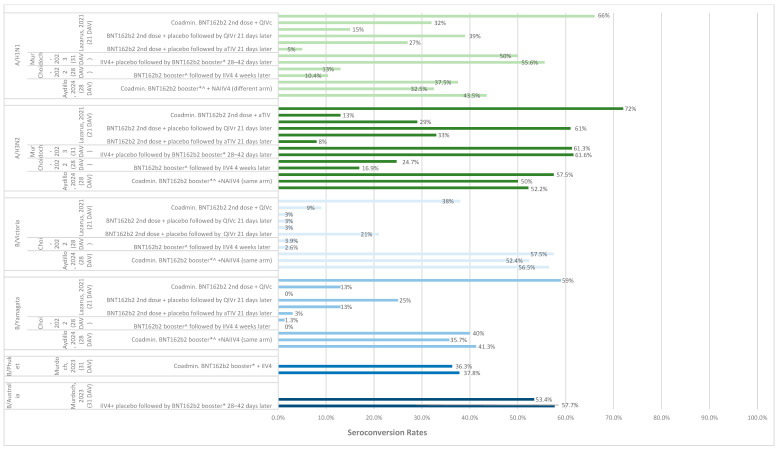
Seroconversion rates of Anti-S IgG. Abbreviations: DAV = days after vaccination; IIV4 = quadrivalent inactivated influenza vaccine; NAIIV4 = quadrivalent non-adjuvanted inactivated vaccine; QIVc = quadrivalent inactivated influenza vaccine (cell-based). * Original monovalent; ^ bivalent BA.5; *^ original monovalent and bivalent BA.4/5 [18,34,35,37].

**Figure A2:**
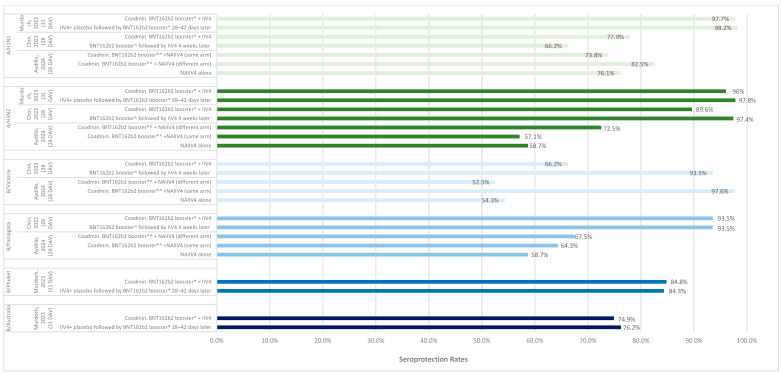
Seroprotection rates against influenza strains A and B. Abbreviations: DAV = days after vaccination; IIV4 = quadrivalent inactivated influenza vaccine; NAIIV4 = quadrivalent non-adjuvanted inactivated vaccine; QIVc = quadrivalent inactivated influenza vaccine (cell-based). * Original monovalent; ^ bivalent BA.5; *^ original monovalent and bivalent BA.4/5 [18,35,37].

Seroconversion rates against influenza A (A/H1N1 and A/H3N2) and B strains (B/Victoria, B/Yamagata, B/Phuket, and B/Australia) 21-, 28-, or 31- days post-vaccination were reported in four studies [18,33,34,37], with three also reporting on seroprotection (Appendix A Figure A1 and Figure A2). Seroconversion rates against various influenza strains varied from 0% to 72%. At 21 days post-vaccination, co-administration of the BNT162b2 second dose and QIVr showed higher seroconversion rates compared to sequential administration with QIVr, QIVc, or IIV3 [34]. Similar results were observed at 28 days with the BNT162b2 bivalent BA.4/5 booster and IIV4 [37]. In contrast, at 28 days, co-administration of the BNT162b2 monovalent booster and IIV4 showed lower seroconversion rates compared to sequential administration [35]. Slightly lower rates were observed with the co-administration of the BNT162b2 monovalent or bivalent BA.4/5 booster and quadrivalent non-adjuvanted inactivated vaccine (NAIIV4; different arm) compared with NAIIV4 alone [18].

The seroprotection was defined as a hemagglutination inhibition (HI) titer of ≥40, as determined by the HI assay [18,35,37]. Seroconversion rate against influenza strain A (A/H1N1 and A/H3N2) and B (B/Victoria, B/Yamagata, B/Phuket, and B/Australia) strains at 31 days was generally lower with co-administration of the BNT162b2 monovalent booster and IIV4 compared to sequential administration 28–42 days later, except for B/Phuket, which showed higher rates in the co-administration group [35]. Similar results were observed at 28 days with the BNT162b2 bivalent BA.4/5 booster and IIV4 compared to the BNT162b2 bivalent BA.4/5 booster followed by IIV4 21 days later, except for A/H1N1 [37]. Conversely, at 28 days, co-administration of the BNT162b2 monovalent or bivalent BA.4/5 booster and NAIIV4 showed higher seroprotection rates compared to NAIIV4 alone [18].

#### 3.3.2. Antibody Response Metrics

One study reported the geometric mean concentrations of IgA and IgG antibodies following various vaccine sequences. Both IgA and IgG responses were similar across sequences at day 0 (~100 BAU/mL). However, the COVID-19 vaccine alone elicited the highest antibody response at day 21 [33]. Co-administration of BNT162b2 plus IIV4 on the same day resulted in the lowest anti-S antibody response at day 21, increasing from 117 to 857 BAU/mL. Another study found a higher geometric mean fold rise 1-month post-vaccination in response to sequential vaccination (SIV followed by BNT162b2 28–48 days later) compared with co-administration of both vaccines on the same day (2.5 vs. 3.1, respectively) [35].

Anti-influenza geometric mean titer (GMT) varied across studies [18,34,37,41], with units often not described. On the day of vaccination, there were no major differences in GMT based on type of SIV (cellular quadrivalent, recombinant quadrivalent, or MF59C adjuvanted trivalent vaccine), SIV alone, or vaccine sequence across the included studies and evaluated strains [34,37,41]. Same-day co-administration of BNT162b2 plus SIV resulted in higher GMTs against all influenza A strains at approximately 21 days following vaccination, compared with sequential vaccination [34]. No differences were seen in antibody responses when testing influenza B strains. One study reported that co-administration in a different arm resulted in a nearly doubled antibody response compared with the same arm for all influenza A strains at day 0 and day 28 following vaccination [18].

#### 3.3.3. Cell-Mediated Immunity

One study evaluated cell-mediated immunity following co-administration of the BNT162b2 booster with NAIIV4 in the same arm vs. different arms and not at staggered time intervals [18]. The percentage of patients with CD4+ T-cells expressing interleukin (IL)-6 at day 7 was higher in the different-arm group compared with the same-arm group (71.1% vs. 39%, *p* = 0.007). No significant difference was observed on day 28 (different-arm: 16.2% vs. same-arm: 41.5%, *p* = 0.020). Similarly, IL-6-expressing monocytes were higher in the group receiving NAIIV4 alone compared with the same-arm group (80% vs. 58.5%, *p* = 0.019) and in the different-arms group compared to the same-arm group (86.8% vs. 58.5%, *p* < 0.01) [18].

No statistically significant differences were reported in CD4+ T-cells (expressing interferon [INF]-ɣ, IL-2, and tumor necrosis factor [TNF]-α), CD8+ T-cells (expressing INF-ɣ, IL-2, IL-6, and TNF-α), and monocytes (expressing INF-ɣ, IL-2, and TNF-α) among the groups receiving the co-administration of the BNT162b2 booster with NAIIV4 in the same arm, the co-administration of the BNT162b2 booster with NAIIV4 in different arms.

### 3.4. Efficacy and Effectiveness of Same-Day Co-Administration

The effectiveness of the co-administration of BNT162b2 with SIVs compared to single administration was evaluated in two studies: one conducted in the US [27] and one in Italy [29]. Efficacy was assessed in one study conducted in Korea [37] (Appendix A Table A4).

In a retrospective study conducted in US on adults 18 years of age and older between August 2022 and January 2023, co-administration of the BNT162b2 BA.4/5 bivalent booster with SIV compared to the BNT162b2 BA.4/5 bivalent booster alone resulted in a similar incidence of influenza-related hospitalizations (0.03% vs. 0.03%, respectively; adjusted hazard ratio [AHR], 0.92; 95% CI, 0.69–1.23), as well as COVID-19-related hospitalizations (0.03% vs. 0.02%, respectively; AHR, 1.55; 95% CI, 0.88–2.73) [27]. The SIVs received included inactivated egg-based SIV (49%), inactivated cell-based SIV (42%), and recombinant SIV (8.2%). There were no statistically significant differences between influenza-related medical encounters; however, the co-administration group reported a higher incidence of COVID-19-related emergency department or urgent care encounters (0.06% vs. 0.04%, respectively; AHR, 1.57; 95% CI, 1.08–2.26) and incidence of outpatient visits (2.06% vs. 1.71%, respectively; AHR, 1.14; 95% CI, 1.07–1.21) at 105 days of follow-up. Among individuals aged 65 years or older, the co-administration group reported a lower incidence of influenza-related hospitalization (0.07% vs. 0.09%, respectively; AHR, 0.83; 95% CI, 0.72–0.95), incidence of outpatient visits (0.45% vs. 0.52%, respectively; AHR, 0.86; 95% CI, 0.81–0.91), and similar incidence of emergency department or urgent care encounters (0.22% vs. 0.23%, respectively; AHR, 0.93; 95% CI, 0.86–1.01) at 105 days of follow-up [27]. Non-statistically significant higher incidence of COVID-19-related hospitalization was reported in older adults receiving co-administered vaccines (0.13% vs. 0.12%, respectively; AHR, 1.04; 95% CI, 0.87–1.24), whereas a statistically significant higher incidence was reported for emergency department or urgent care encounters (0.50% vs. 0.42%, respectively; AHR, 1.12; 95% CI, 1.02–1.23) and outpatient visits (1.94% vs. 1.76%, respectively; AHR, 1.14; 95% CI, 1.01–1.11) at 150 days of follow-up [27].

A prospective study conducted in Italy among healthcare workers (age range: 20–78 years) from 12 to 22 October 2021 reported the incidence of SARS-CoV-2 infection following different vaccination strategies. The highest incidence of SARS-CoV-2 infection was observed in those receiving the BNT162b2 monovalent booster alone (44.96 per 100 subjects; 95% CI, 38.64–51.28), followed by those who received the quadrivalent inactivated influenza cell-based vaccine (QIVc) with a delayed BNT162b2 monovalent booster until after 22 October 2021 (42.55 per 100 subjects; 95% CI, 32.56–52.55), and lowest in those receiving co-administration of the BNT162b2 monovalent booster with QIVc (40.00 per 100 subjects; 95% CI, 36.11–43.89) during the 280-day follow-up. No statistically significant differences in the incidence of symptomatic SARS-CoV-2 infection were observed between groups [29].

Lastly, in a non-randomized trial of healthy adults (age range: 20–64 years) in Korea conducted between October and December 2022, one case of influenza infection was reported in a group of 77 individuals who received co-administration of BNT162b2 BA.4/5 bivalent booster with quadrivalent inactivated influenza vaccine (IIV4) (1.3%). No cases of influenza were reported in the group that received both vaccines separately [37]. No cases of COVID-19 infection were reported following the co-administration of BNT162b2 BA.4/5 bivalent booster with IIV4 or separately.

### 3.5. Safety/Reactogenicity of Same-Day Co-Administration

**Figure 4:**
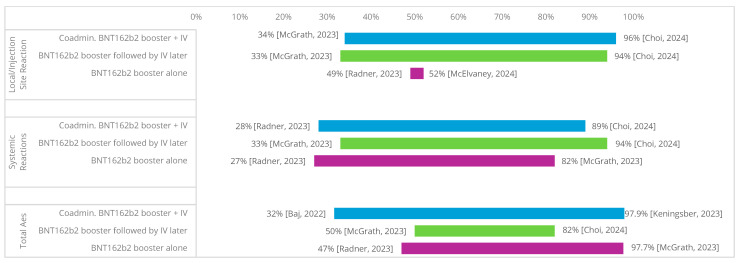
Safety/reactogenicity outcome ranges. Abbreviations: Ae = adverse event, IV = influenza vaccine [19,23,26,27,31,37].

The safety and/or reactogenicity of same-day co-administration of BNT162B2 and SIV were reported in 12 studies (Figure 4, Appendix A Table A5). Six of the studies were conducted in general adult populations; four studies were conducted specifically in healthcare workers, one in an elderly population, and one in an immunocompromised population. The administration of the BNT162b2 second dose was reported in 1 study, whereas the administration of the BNT162b2 booster was reported in 11 studies. Of these, 25% specifically reported on the BNT162b2 BA.4/5 bivalent booster (n = 3). Among the SIV evaluated, egg-based IIV4 was the most commonly administered in eight studies, including in one study with patients over 60 years old [33]. In contrast, QIVc (n = 4), aTIV (n = 1), and QIVr (n = 1) were less commonly used.

Ten studies reported data on local or injection site reactions [20,22,26,29,32,33,34,35,36,37]. Total local or injection site reaction rates for same-day co-administration of the BNT162b2 booster with influenza vaccination ranged from 34% [26] to 96% [34], BNT162b2 booster followed by SIV later ranged from 33% [26] to 94% [34], and BNT162b2 booster alone ranged from 49% [20] to 52% [36]. In adults aged 60 and older, slightly increased rates of pain (86.8%) were present among those receiving co-administered BNT162b2 and egg-based IIV4 compared with sequential vaccination (IIV4 first: 21.1%; BNT162b2 first: 63.2%) or BNT162b2 alone (71.1%). This group experienced a similar or reduced incidence of swelling and redness at the injection site [33].

Nine studies reported data on systemic reactions [20,22,26,32,33,34,35,36,37]. Total systemic reaction rates for co-administration of the BNT162b2 booster with SIV ranged from 28% [20] to 89% [34], BNT162b2 booster followed by SIV later ranged from 33% [26] to 94% [34], and BNT162b2 booster alone ranged from 27% [20] to 82% [26]. Four studies [32,33,35,36] reported individual local or injection site reactions. In adults aged 60 and older, fatigue, myalgia, joint pain, and headache were more frequent among those with co-administered vaccines [33].

Five studies reported data on adverse events [19,29,31,35,36]. The total adverse events rates for co-administration of the BNT162b2 booster with SIV ranged from 31.6% [35] to 97.9% [31], BNT162b2 booster followed by SIV later ranged from 50% [26] to 82% [34], and BNT162b2 booster alone ranged from 47% [20] to 97.7% [26]. Immunocompromised individuals did not have a higher rate of adverse events compared with the general population [26].

## 4. Discussion

This systematic review included 20 studies examining the co-administration of BNT162b2 with SIV in both real-world and clinical trial settings. These studies involved diverse populations across multiple countries, including the US, UK, Netherlands, Austria, Australia, New Zealand, Korea, Spain, Ireland, Israel, and Italy. Furthermore, the data captured co-administration of BNT162B2 with different types of IV across two influenza seasons. Overall, this systematic review found that co-administering BNT162b2 with SIV is safe and does not have a significant impact on the effectiveness of these vaccines. This review, therefore, supports the notion that co-administration can reduce the number of healthcare visits and missed opportunities to vaccinate, which may improve vaccine uptake.

Data included in this review indicated that the prevalence of reported co-administration was higher in 2022 than in 2021, signaling an increasing trend in the co-administration of influenza and the BNT162B2 vaccine. The WHO and Centers for Disease Control and Prevention, among other health authorities, have endorsed the simultaneous administration of these vaccines, highlighting their potential to enhance vaccination coverage and streamline healthcare delivery [12,13]. These recommendations, coupled with the convenience of receiving both vaccines in a single visit for both patients and healthcare providers, have likely contributed to the increased adoption of this practice [1]. The relatively low prevalence of co-administration observed in the earlier part of the COVID-19 pandemic in the included studies likely reflects operational factors such as the physical separation of vaccine deployment sites early in the COVID-19 pandemic and evolving co-administration recommendations [13,42,43,44].

Furthermore, this review found that the practice of co-administration was higher among healthcare workers compared with the general population. The increased adoption of co-administration practices, particularly among healthcare workers, may reflect both logistical efficiencies and occupational health priorities aimed at reducing transmission risks in healthcare settings [45,46]. Protecting healthcare workers is critical to maintaining a resilient healthcare system, as noted in infection prevention frameworks that prioritize vaccination as a cornerstone of occupational health strategies [47].

Immunogenicity, as established in prior literature, is a key determinant of vaccine efficacy and effectiveness [48]. Updates to both COVID-19 vaccines and SIV to address emerging variants and seasonally circulating strains may lead to differences in immunogenicity profiles, making it challenging to draw definitive conclusions from existing immunogenicity studies. Overall, no consistent pattern indicating a negative impact on immunogenicity from same-day co-administration was identified. Although specific immunogenicity outcomes varied with different vaccination strategies, vaccine performance in preventing clinical outcomes demonstrated comparability between co-administered and individually administered vaccine groups, which is the primary consideration for public health [12,13]. Additionally, adverse events, both local and systemic, were comparable across co-administration, sequential, and single vaccination groups. This aligns with the results from existing reviews on the co-administration of SIV and COVID-19 vaccines, supporting co-administration as a feasible and safe approach, especially in high-demand healthcare settings, without negatively impacting patient outcomes or vaccine effectiveness [1,11]. Co-administration of vaccines offers numerous logistical, clinical, and public health advantages [21]. It reduces the number of healthcare visits, minimizing the risk of attrition associated with multiple visits, thus potentially increasing the uptake of both vaccine components [21]. Additionally, pairing vaccines shortens the time needed to achieve protection against co-circulating viruses and reduces the disease burden [21]. Overall, the evidence supports the co-administration of BNT162B2 with SIV as a practical approach to seasonal respiratory vaccination, allowing for efficient resource utilization without compromising vaccine effectiveness or safety.

A key strength of this review is the inclusion of both clinical trial data and observational data, providing a comprehensive perspective on co-administration across diverse, real-world settings and populations. This is the first systematic review to incorporate both types of data, which enhances the generalizability of findings and strengthens the evidence base for co-administration of BNT162B2 as a viable public health strategy. However, it is important to recognize the inherent limitations of observational studies, which can be more susceptible to biases and confounding than randomized, controlled trials. Nonetheless, the inclusion of both RCTs and moderately robust observational studies strengthens the evidence base by balancing the observational data with more controlled clinical evidence. The included RCTs generally showed a low risk of bias, particularly in randomization, outcome data, and result reporting, though some domains had unclear levels of bias due to limited blinding details. Included observational studies displayed a moderate risk of bias, primarily due to variability in adjustments for confounding factors, though most accounted for key variables.

Results from this review must be interpreted considering several limitations. For one, the heterogeneity across studies in terms of study designs, methodologies, study populations, circulating virus strains, and vaccine formulations precluded the meta-analysis of results and may affect the comparability of findings. While the inclusion of various SIV platforms induces heterogeneity, it also reflects real-world immunization practices. This variability should be considered when interpreting findings, particularly in outcomes supported by a limited number of studies. Such variability is common in vaccine research due to differences in regional public health practices and healthcare infrastructure [49]. This review included only studies with comparable endpoints and outcomes, which helped to address this limitation where possible. Moreover, there is a paucity of data on long-term outcomes and the durability of immune responses in co-administered vaccine recipients. Although shorter-term studies provide substantial evidence of co-administration’s immediate safety and effectiveness, additional long-term studies could provide insights into the durability of immune responses. Such research would complement current evidence.

## 5. Conclusions

The evidence supports the safety and effectiveness of co-administering BNT162B2 with SIV in adults ≥ 18 years of age. This review contributes to the robust foundation of evidence that exists to support the recommendation of co-administration strategies for SIV and COVID-19 vaccines to optimize immunization efforts while addressing the inherent challenges of heterogeneity and design variability.

## Figures and Tables

**Table 1:** PICOS eligibility criteria.

Domain	Inclusion Criteria	Exclusion Criteria
Population	General population ≥ 18 years of age, including high-risk groups (for either/or influenza and COVID-19), receiving COVID-19 and influenza vaccinations Population subgroups of interest include ages 18–49, 50–64, and ≥65 years, and other age groups reported in the literature	Population < 18 years of age Studies based on the general population with no reported COVID-19 or flu vaccination Age information not available
Intervention **	To inform the efficacy, effectiveness, safety, and immunogenicity outcomes, co-administration of BNT162b2 (any formulation, including original monovalent, bivalent, and XBB.1.5-adapted) and any type of SIV (and valency, including both TIV and QIV) will be defined as on the same medical/pharmacy visit or day To inform the prevalence of administration, co-administration will first be defined as vaccines of interest administered within 3 months of each other with or without any third vaccine (e.g., pneumococcal vaccine, RSV) For all outcomes, booster refers to any BNT162b2 vaccination other than the primary two-dose series	COVID-19 vaccines other than BNT162b2 Co-administration of BNT162b2 with no vaccine against influenza ^a^ Vaccines where the time interval between co-administration is not reported
Comparator	Any other approved vaccine (e.g., BNT162b2 or other COVID-19 vaccination alone, SIV alone, RSV vaccine alone) No vaccination or no recent vaccination None required (for non-vaccine efficacy/effectiveness evidence)	NA
Outcomes	Outcomes including Prevalence: Prevalence of co-administration by time interval Factors influencing co-administration, if reported (e.g., public health guidelines, vaccine availability, healthcare provider recommendation) Efficacy/Effectiveness: SIV: Influenza-like infection (ILI) Influenza-related medical encounters (including outpatient, urgent care, and ER visits) Laboratory-confirmed influenza cases with associated ILI Hospitalizations for influenza Influenza-related ICU admission Influenza-related mortality Hospitalization for pneumonia Influenza-related medical encounters (including outpatient, urgent care, and ER visits) BNT162b2 vaccine: Laboratory-confirmed SARS-CoV-2 infection (regardless of presence of symptoms) Laboratory-confirmed symptomatic COVID-19 Laboratory-confirmed severe COVID-19 Laboratory-confirmed critical COVID-19 COVID-19-like infection COVID-19-related medical encounters (including outpatient, urgent care, and ER visits) COVID-19-related Hospitalizations COVID-19-related mortality COVID-19-related ICU admission Hospitalization for pneumonia Immunogenicity: Seroconversion Seroprotection GMTs for HI GMCs for anti-spike antibodies Cell-mediated immunity Safety/Reactogenicity: Total AEs Total SAEs Total grade 3+ treatment-related AEs Total AESIs Any SAEs leading to hospitalization Cardioembolic events Injection site reaction	Publications that do not report on at least one of the outcomes listed in the inclusion criteria
Study Design	Clinical trials phases 1–3 (randomized/non-randomized) Post-hoc analysis of trials Pooled analysis of trials Observational studies, including prospective/retrospective cohort studies, case–control studies (including case-only designs), ecological studies	SLRs/MA ^b^ Pharmacodynamic/pharmacokinetic studies Genetic studies or cellular/molecular studies Trials without results Trial protocols Case reports, case studies, or case series Narrative reviews Editorials, theses, dissertations, book chapters, news articles
Publication Type	Full-text publications, including preprints posted within 6 months of the search date Conference abstracts Conference posters/presentations (where available) Letters to the editor, comments, commentaries	Preprints older than 6 months ^c^
Publication Dates	2021–7 August 2024	Publications indexed before 1 January 2021
Language	English language	Non-English-language publications
Geography	No geographic limits	Not applicable

^a^ Interventions that involve the co-administration of BNT162b2 with any other approved vaccine were tagged. ^b^ The relevant analyses/reviews identified in the database searches were used only for bibliography checking; however, to avoid potential double-counting of data, they were not formally included in the SLR. ^c^ A date limit was used to restrict the inclusion of preprints into the literature review. All preprints were flagged as such in the study listing, with any older than 6 months excluded. ** Studies that involve co-administration of IV with COVID-19 vaccines other than BNT162B2 were flagged. Abbreviations: AE = adverse event; AESI = adverse event of special interest; AR = adverse reaction; ER = emergency room; GMC = geometric mean concentrations; GMT = geometric mean titer; HI = hemagglutinin inhibition; ICU = intensive care unit; ILI = influenza-like infection; MA = meta-analysis; PICOS = population, interventions, comparisons, outcomes, and study design; QIV = quadrivalent influenza vaccine; RCT = randomized controlled trial; RQ = research question; RSV = respiratory syncytial virus; SAE = serious adverse event; SIV = seasonal influenza vaccine; SLR = systematic literature review; TIV = trivalent influenza vaccine.

## Data Availability

Not applicable.

## Appendix A

**Table A1:** Study characteristics of included studies.

Author, Year	Study Design	Population Description	Country; Center/Setting	Sample Size	Intervention	Comparator	Length of Follow-Up (Days)	Prevalence	Efficacy/ EFF	IG	Safety/ RTG
Lazarus, 2021 [34]	RCT	General population	UK Multi-center	679	1. Coadmin. BNT162b2 second dose with QIVc (Flucelvax, Seqirus) same day 2. Coadmin. BNT162b2 second dose with MF59C aTIV (FluAd, Seqirus, Maidenhead), same day 3. Coadmin. BNT162b2 second dose with QIVr (Flublok, Sanofi), same day	1. BNT162b2 second dose with placebo followed by MF59C aTIV (FluAd-Seqirus, Maidenhead) 21 days later 2. BNT162b2 second dose with placebo followed by QIVc (Flucelvax, Seqirus) 21 days later 3. BNT162b2 second dose with placebo followed by QIVr (Flublok, Sanofi) 21 days later	42 days	No	No	Yes	Yes
Dulfer, 2023 [33]	RCT	Patients ≥60 years of age	Netherlands Single center	154	1. Coadmin. BNT162b2 booster with IIV4 (Vaxigrip Tetra, Sanofi), same day	1. IIV4 (Vaxigrip Tetra, Sanofi) with placebo followed by BNT162b2 booster 21 days later 2. BNT162b2 booster with placebo followed by IIV4 (Vaxigrip Tetra), Sanofi 21 days later 3. BNT162b2 booster with placebo followed by placebo 21 days later	Visits each 21 days apart	No	No	Yes	Yes
Murdoch, 2023 [35]	RCT	General population	Australia and New Zealand Multi-center	564	1. Coadmin. BNT162b2 second booster with IIV4 (Afluria Quad, Seqirus), same day	1. IIV4 (Afluria Quad, Seqirus) with placebo followed by BNT162b2 second booster 28–42 days later	30 days	No	No	Yes	Yes
Ramsay, 2023 [36]	RCT	General population	Australia Multi-center	71	1. Coadmin. BNT162b2 first booster with SIV (including Fluarix Tetra, GlaxoSmithKline, FluQuadri, Sanofi, Fluad Quad, Seqirus), same day 2. Coadmin. BNT162b2 first booster (fourth dose) with SIV (including Fluarix Tetra, GlaxoSmithKline, FluQuadri, Sanofi, Fluad Quad, Seqirus), same day	1. BNT162b2 first booster with placebo followed by SIV (including Fluarix Tetra, GlaxoSmithKline, FluQuadri, Sanofi, Fluad Quad, Seqirus) 7–14 days later 2. BNT162b2 second booster with placebo followed by SIV (including Fluarix Tetra, GlaxoSmithKline, FluQuadri, Sanofi, Fluad Quad, Seqirus) 7–14 days later	7 days	No	No	No	Yes
Choi, 2024 [37]	Non-randomized trials	General population	Korea Single center	77	1. Coadmin. BNT162b2 bivalent BA.5 second booster with IIV4 (GC flu, GC Biopharma Corp), same day	1. BNT162b2 bivalent BA.5 second booster followed by IIV4 4 weeks later	28 days	No	Yes	Yes	Yes
Aydillo, 2024 [18]	Observational, Prospective	General population	US, Spain Multi-center	128	1. Coadmin. BNT162b2 booster with NAIIV4 (QIV, Sanofi Pasteur), same arm in the same day 2. Coadmin. BNT162b2 booster with NAIIV4 (QIV, Sanofi Pasteur), different arm on the same day	1. NAIIV4 alone	28 days	No	No	Yes	No
Harris, 2024 [21]	Observational, Cross-sectional	Medicare beneficiaries ≥66 years of age	US Multi-center	Period 1: 6,292,777 Period 2: 4,757,501	1. Coadmin. BNT162b2 booster with SIV (Fluzone High-Dose Quad, Sanofi Pasteur, FluMist Quadrivalent, AstraZeneca, Flucelvax Quadrivalent, Seqirus, Flublok Quadrivalent, Sanofi Pasteur, Fluad Trivalent, CSL Seqirus), same day	NA	7	Yes	No	No	No
Lu, 2024 [25]	Observational, Case series	Patients ≥65 years of age	US Multi-center	9,040,176	1. Coadmin. BNT162b2 bivalent BA.4/5 booster with QIV (Fluzone, Sanofi Pasteur, Fluad, Seqirus), same day	NA	90 days	Yes	No	No	No
McElvaney, 2024 [26]	Observational, Prospective	Patients with alpha-1 antitrypsin deficiency	Ireland Multi-center	170	1. Coadmin. BNT162b2 BA.4/5 bivalent second booster with QIV (NR), same day	NA	2 to 8 days	No	No	No	Yes
Gonen, 2023 [20]; Moss, 2023 [41]	Observational, Prospective	General population	Israel Single center	649	1. Coadmin. BNT162b2 BA.4/5 bivalent booster with IIV4 (Influvac Tetra, Abbott), same day	1. BNT162b2 BA.4/5 bivalent booster alone 2. IIV4 alone	60 days	No	No	Yes	Yes
Kenigsberg, 2023 [23]	Observational, Retrospective	General population	US Multi-center	2,301,876	1. Coadmin. BNT162b2 BA.4/5 bivalent booster with QIV (NR), same day	NA	NR	Yes	No	No	No
Kim, 2023 [24,39]	Observational, Prospective	Healthcare workers	Korea Single center	2061	1. IIV4 (Boryung FLU Vaccine VIII-TFinj^®^, Boryung Biopharma) followed by BNT162b2 first booster 1 week later	NA	7 days	Yes	No	No	No
McGrath, 2023 [27]	Observational, Retrospective	General population	US Multi-center	3,442,996	1. Coadmin. BNT162b2 BA.4/5 bivalent booster + SIV (Flublok, Sanofi Pasteur; Fluzone, Sanofi Pasteur, Fluad, Seqirus, Flucelvax, Seqirus, FluMist, AstraZeneca), same day	1. BNT162b2 BA.4/5 bivalent booster alone 2. SIV alone (Flublok, Sanofi Pasteur, Fluzone, Sanofi Pasteur, Fluad, Seqirus, Flucelvax, Seqirus, FluMist, AstraZeneca)	Median: 109 days (IQR, 89–125) for the co-administration of BNT162b2-biv and SIV Median: 51 days (IQR, 17–99) for the BNT162b2-biv alone Median: 90 days (IQR, 49–112) for SIV alone	Yes	Yes	No	No
Moro, 2023 [28]	Observational, Retrospective	General population	US Multi-center	2449	1. Coadmin. BNT162b2 first booster with SIV (Fluzone, Sanofi Pasteur, Afluria, Seqirus, Fluzone high dose, Sanofi Pasteur), same medical visit	NA	NR	Yes	No	No	No
Moscara, 2023 [29]	Observational, Prospective	Healthcare workers	Italy Single center	942	1. Coadmin. BNT162b2 booster with QIVc (Flucelvax Tetra, Seqirus), same day	1. BNT162b2 booster alone 2. QIVc followed by BNT162b2 booster after 22 October 2021	280 days	No	Yes	No	Yes
Pascucci, 2023 [30,38]	Observational, Retrospective	Healthcare workers	Italy Single center	7399	1. Coadmin. BNT162b2 BA.4/5 bivalent booster with QIV (Vaxigrip Tetra, Sanofi Pasteur, Flucelvax Tetra, Seqirus), same day	NA	NR	Yes	No	No	No
Radner, 2023 [31]	Observational, Prospective	Healthcare workers	Austria Multi-center	838	1. Coadmin. BNT162b2 first booster with QIV (Vaxigrip Tetra, Sanofi Pasteur, Flucelvax Tetra, Seqirus), same day	1. BNT162b2 first booster alone 2. QIV (Vaxigrip Tetra, Sanofi Pasteur, Flucelvax Tetra, Seqirus) alone	28 days	No	No	No	Yes
Baj, 2022 [19]	Observational, Prospective	Healthcare workers	Italy Single center	64	1. Coadmin. BNT162b2 first booster with IIV4 (Vaxigrip Tetra, Sanofi), same day	NA	14 days	No	No	No	Yes
Hause, 2022 [22]	Observational, Retrospective	General population	US NR	61,390	1. Coadmin. BNT162b2 first booster with IV (NR), same day	1. BNT162b2 first booster alone	0 to 7 days post-vaccination	Yes	No	No	Yes
Venuto, 2022 [32]	Observational, Retrospective	Healthcare workers	Italy Single center	64	1. Coadmin. BNT162b2 first booster + QIV (Vaxigrip Tetra, Sanofi, Flucelvax Tetra, Seqirus), same day	1. BNT162b2 first booster alone	30 days	No	No	No	Yes

Abbreviations: aTIV = adjuvanted trivalent influenza vaccine; EFS = effectiveness; IG = immunogenicityIIV4 = quadrivalent inactivated influenza vaccine; IV = influenza vaccine; QIV = quadrivalent influenza vaccine; QIVc = quadrivalent inactivated influenza vaccine (cell-based), QIVr = quadrivalent influenza vaccine, recombinant; RTG = reactogenicity; SIV = seasonal influenza vaccine.

**Table A2:** Cochrane risk of bias assessment for randomized controlled trials.

Trial, Reference	Bias From					Overall Bias
	Randomization Process	Deviations From Intended Interventions	Missing Outcome Data	Measurement of the Outcome	Selection of the Reported Result	
Dulfer, 2023 [33]						
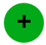
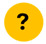
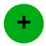
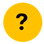
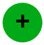
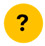
Ramsay, 2023 [36]						
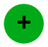
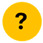
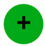
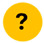
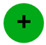
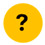
Lazarus, 2021 [34]						
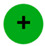
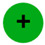
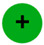
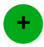
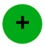
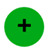
Murdoch, 2023 [35]						
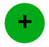
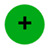
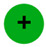
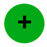
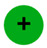
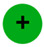

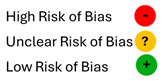
	

**Table A3:** Newcastle–Ottawa risk of bias assessment for observational studies and non-randomized trials.

Trial, Reference	Bias From			Overall Bias
	Selection	Comparability	Outcome	
Harris, 2024 [21]	3	0	3	6
Baj, 2022 [19]	3	0	3	6
Gonen, 2023 [20]	3	0	3	6
Choi, 2024 [37]	3	0	3	6
Hause, 2022 [22]	4	1	2	7
Kenigsberg, 2023 [23]	4	0	3	7
Kim, 2023 [24]	3	0	3	6
Lu, 2024 [25]	3	0	3	6
McElvaney, 2024 [26]	3	0	3	6
McGrath, 2023 [27]	4	0	3	7
Aydillo, 2024 [18]	3	0	3	6
Moro, 2023 [28]	3	0	3	6
Moscara, 2023 [29]	3	1	3	7
Venuto, 2022 [32]	3	0	3	6
Pascucci, 2023 [30]	3	0	3	6
Radner, 2023 [31]	3	0	3	6

**Table A4:** Efficacy and effectiveness of same-day co-administration.

**Outcomes**	**Moscara, 2023** [29]			**McGrath, 2023** [27]			**Choi, 2024** [37]	
	**Coadmin. BNT162b2 Booster with QIV, Same Day**	**BNT162b2 Booster Alone**	**QIVc Followed by BNT162b2 Booster after the 22nd of October 2021**	**Coadmin.BNT162b2 BA.4/5 Bivalent Booster with SIV, Same Day**	**BNT162b2 BA.4/5 Bivalent Booster Alone**	**SIV Alone**	**Coadmin. BNT162b2 Bivalent BA.5 Second Booster with IIV4, Same Day**	**BNT162b2 Bivalent BA.5 Second Booster Followed by IIV4 4 Weeks Later**
	***Influenza vaccines***							
Influenza-like infection (ILI), n (%)	NR	NR	NR	NR	NR	NR	1 (1.3%)	0 (0)
Hospitalizations for influenza	NR	NR	NR	18 to 64 years: 0.03% ≥65 years: 0.07%	NR	18 to 64 years: 0.03% ≥65 years: 0.09%	NR	NR
Influenza-related medical encounters (including emergency department or urgent care), %	NR	NR	NR	18 to 64 years: 0.09% ≥65 years: 0.22%	NR	18 to 64 years: 0.08% ≥65 years: 0.23%	NR	NR
Influenza-related medical encounters (including outpatient visits), %	NR	NR	NR	18 to 64 years: 0.70% ≥65 years: 0.45%	NR	18 to 64 years: 0.94% ≥65 years: 0.52%	NR	NR
	***BNT162b2 vaccines***							
Laboratory-confirmed SARS-CoV-2 infection (regardless of presence of symptoms)	40.00 per 100 subjects (95% CI: 36.11–43.89)	44.96 per 100 subjects (95% CI: 38.64–51.28)	42.55 per 100 subjects (95% CI: 32.56/52.55)	NR	NR	NR	NR	NR
Laboratory-confirmed symptomatic COVID-19	32.62 per 100 subjects (95% CI: 28.90–36.34)	37.82 per 100 subjects (95% CI: 31.65–43.98)	36.17 per 100 subjects (95% CI: 26.46–48.88)	NR	NR	NR	NR	NR
COVID-19--like infection (CLI), n (%)	NR	NR	NR	NR	NR	NR	0 (0)	0 (0)
COVID-19-related hospitalizations, %	NR	NR	NR	18 to 64 years: 0.03% ≥65 years: 0.13%	18 to 64 years: 0.02% ≥65 years: 0.12%	NR	NR	NR
COVID-19-related medical encounters (including emergency department or urgent care), %	NR	NR	NR	18 to 64 years: 0.06% ≥65 years: 0.50%	18 to 64 years: 0.04% ≥65 years: 0.42%	NR	NR	NR
COVID-19-related medical encounters (including outpatient visits), %	NR	NR	NR	18 to 64 years: 2.06% ≥65 years: 1.94%	18 to 64 years: 1.71% ≥65 years: 1.76%	NR	NR	NR

**Table A5:** Safety/Reactogenicity Outcomes.

Author, Year	Population Description	Treatment Name	Sample Size	Timepoint	Local/Injection Site Reaction, n (%)	Systemic Reactions, n (%)	Total AEs, n (%)
Baj, 2022 [19]	Healthcare workers	Coadmin. BNT162b2 booster + IIV4	36	14 days after vaccination	NR	NR	18 (50.0)
		BNT162b2 booster alone	28		NR	NR	20 (71.0)
Choi, 2024 [37]	General population	Coadmin. BNT162b2 BA.4/5 bivalent booster + IIV4	77	7 days after vaccination	36 (46.8)	47 (61.0)	NR
		BNT162b2 BA.4/5 bivalent booster + IIV4 at least 4 weeks apart	77		43 (55.8)	50 (64.9)	NR
Dulfer, 2023 [33]	Patients ≥60 years of age	Coadmin. BNT162b2 booster + IIV4	38	14 days after vaccination	Redness at injection site: 5 (13.2) Pain at injection site: 33 (86.8) Swollen at injection site: 3 (7.9)	Fever: 1 (2.6) Fatigue: 9 (23.7) Myalgia: 13 (34.2) Joint pain: 7 (18.4) Headache: 12 (31.6) Chills: 5 (13.2) Nausea: 0 (0.0)	NR
		IIV4 + placebo followed by BNT162b2 booster 21 days later	38		Redness at injection site: 3 (7.9) Pain at injection site: 8 (21.1) Swollen at injection site: 1 (2.6)	Fever: 0 (0.0) Fatigue: 7 (18.4) Myalgia: 3 (7.9) Joint pain: 4 (10.5) Headache: 8 (21.1) Chills: 2 (5.3) Nausea: 0 (0.0)	NR
		BNT162b2 booster + placebo followed by IIV4 21 days later	38		Redness at injection site: 5 (13.2) Pain at injection site: 24 (63.2) Swollen at injection site: 9 (23.7)	Fever: 2 (5.3) Fatigue: 8 (21.1) Myalgia: 7 (18.4) Joint pain: 3 (7.9) Headache: 6 (15.8) Chills: 4 (10.5) Nausea: 1 (2.6)	NR
		BNT162b2 booster + placebo followed by placebo 21 days later	38		Redness at injection site: 3 (7.9) Pain at injection site: Swollen at injection site: 4 (10.5)	Fever: 3 (7.9) Fatigue: 6 (15.8) Myalgia: 10 (26.3) Joint pain: 3 (7.9) Headache: 9 (23.7) Chills: 5 (13.2) Nausea: 3 (7.9)	NR
Gonen, 2023 [20]	General population	Coadmin. BNT162b2 BA.4/5 bivalent booster + IIV4	146	Mean 28.9 days after vaccination	76 (52.0)	40 (28.0)	NR
		BNT162b2 booster alone	85		42 (49.0)	23 (27.0)	NR
Hause, 2022 [22]	General population	Coadmin. BNT162b2 booster + influenza vaccine	61,390	7 days after vaccination	39,818 (64.9)	422,637 (58.9)	NR
		BNT162b2 booster alone	466,439		298,529 (64.0)	274,539 (58.9)	NR
Lazarus, 2021 [34]	General population	BNT162b2 2nd dose + placebo followed by QIVc 21 days later	71	7 days after vaccination	67 (94)	54/67 (81)	NR
		Coadmin. BNT162b2 2nd dose + QIVc	68		65 (96)	59 (87)	NR
		BNT162b2 2nd dose + placebo followed by MF59C aTIV 21 days later	38		30 (79)	25/35 (71)	NR
		Coadmin. BNT162b2 2nd dose + MF59C aTIV	41		31 (76)	24 (59)	NR
		BNT162b2 2nd dose + placebo followed by QIVr 21 days later	29		24/27 (89)	23/28 (82)	NR
		Coadmin. BNT162b2 2nd dose + QIVr	29		25/26 (96)	24/27 (89)	NR
McElvaney, 2024 [26]	Patients with alpha-1 antitrypsin deficiency	Coadmin. BNT162b2 BA.4/5 bivalent booster + QIV	44	8 days after vaccination	15 (34.0)	22 (50.0)	NR
		BNT162b2 BA.4/5 bivalent booster followed by QIV one week later	40		13 (33.0)	20 (50)	
Moscara, 2023 [29]	Healthcare workers	Coadmin. BNT162b2 booster + QIVc	610	7 days after vaccination	NR (57.0)	NR	474 (78.0)
		BNT162b2 booster alone	238		NR (52.0)	NR	190 (80.0)
		QIVc followed by BNT162b2 booster	94		NR (43.0)	NR	53 (56.0)
Murdoch, 2023 [35]	General population	Coadmin. BNT162b2 booster + IIV4	564	7 days after vaccination	Redness: NR (31.6) Swelling: NR (6.2) Pain at injection site: NR (86.2)	Fatigue: NR (64.0) Headache: NR (47.2) Chills: NR (19.9) New or worsened muscle pain: NR (27.7)	NR (31.6) *
		IIV4 + placebo followed by BNT162b2 booster 28–42 days later	564		Redness: NR (29.0) Swelling: NR (0.4) Pain at injection site: NR (13.9)	Fatigue: NR (42.1) Headache: NR (34.3) Chills: NR (6.2) New or worsened muscle pain: NR (11.4)	NR (29.0) *
Radner, 2023 [31]	Healthcare workers	Coadmin. BNT162b2 booster + QIV	240	7 days after vaccination	NR	NR	235 (97.9)
		BNT162b2 booster alone	558		NR	NR	525 (97.9)
		QIV alone	33		NR	NR	26 (78.8)
Ramsay, 2023 [36]	General population	Coadmin. BNT162b2 first booster + SIV	70	7 days after vaccination	Pain at COVID-19 injection site: 29 (41) Pain at SIV/placebo injection site: 5 (7) Swelling at COVID-19 injection site: 2 (3) Swelling at SIV/placebo injection site: 0 (0) Redness at COVID-19 injection site: 0 (0) Redness at SIV/placebo injection site: 0 (0)	Headache: 13 (19) Fatigue: 27 (39) Chills: 5 (7) Myalgia: 13 (19) Joint pain: 5 (7) Nausea: 2 (3) Diarrhea: 0 (0) Fever: 8 (11)	45/71 (63)
		BNT162b2 first booster+ placebo	72		Pain at COVID-19 injection site: 25 (35) Pain at SIV/placebo injection site: 1 (1) Swelling at COVID-19 injection site: 2 (3) Swelling at SIV/placebo injection site: 1 (1) Redness at COVID-19 injection site: 1 (1) Redness at SIV/placebo injection site: 0 (0)	Headache: 15 (21) Fatigue: 22 (31) Chills: 6 (8) Myalgia: 15 (21) Joint pain: 7 (10) Nausea: 5 (7) Diarrhea: 1 (1) Fever: 12 (17)	36/76 (47)
		Coadmin. BNT162b2 second booster + SIV	22		Pain at COVID-19 injection site: 4 (18) Pain at SIV/placebo injection site: 2 (9) Swelling at COVID-19 injection site: 0 (0) Swelling at SIV/placebo injection site: 0 (0) Redness at COVID-19 injection site: 0 (0) Redness at SIV/placebo injection site: 0 (0)	Headache: 3 (14) Fatigue: 5 (23) Chills: 0 (0) Myalgia: 1 (5) Joint pain: 3 (14) Nausea: 0 (0) Diarrhea: 0 (0) Fever: 1 (5)	8/23 (35)
		BNT162b2 second booster + placebo	21		Pain at COVID-19 injection site: 6 (29) Pain at SIV/placebo injection site: 0 (0) Swelling at COVID-19 injection site: 0 (0) Swelling at SIV/placebo injection site: 0 (0) Redness at COVID-19 injection site: 0 (0) Redness at SIV/placebo injection site: 0 (0)	Headache: 3 (14) Fatigue: 5 (24) Chills: 1 (5) Myalgia: 0 (0) Joint pain: 0 (0) Nausea: 0 (0) Diarrhea: 1 (5) Fever: 1 (5)	11/21 (52)
Venuto, 2022 [32]	Healthcare workers	Coadmin. BNT162b2 booster + QIV	64	NR	Local pain: NR (50.6)	Headache: NR (18.8) Chills: NR (8.7) Myalgia: NR (17.3) Joint pain: NR (11.5) Lethargy: NR (7.2) Fever: NR (27.4)	NR
		BNT162b2 booster alone	64		Local pain: NR (53.1)	Headache: NR (17.2) Chills: NR (6.5) Myalgia: NR (15.2) Joint pain: NR (13.3) Lethargy: NR (6.8) Fever: NR (24.0)	NR

* 1 month after vaccination; Abbreviations: AE = adverse event; aTIV = adjuvanted trivalent influenza vaccine; IIV4 = quadrivalent inactivated influenza vaccine; QIV = quadrivalent influenza vaccine; QIVc = quadrivalent inactivated influenza vaccine (cell-based), QIVr = quadrivalent influenza vaccine, recombinant; SIV = seasonal influenza vaccine.

## References

[1] Bonanni P., Steffen R., Schelling J., Balaisyte-Jazone L., Posiuniene I., Zatoński M., Van Damme P. (2023). Vaccine co-administration in adults: An effective way to improve vaccination coverage. Hum. Vaccines Immunother..

[2] Privor-Dumm L.A., Poland G.A., Barratt J., Durrheim D.N., Knoll M.D., Vasudevan P., Jit M., Bonvehí P.E., Bonanni P. (2021). A global agenda for older adult immunization in the COVID-19 era: A roadmap for action. Vaccine.

[3] Lin C.J., Nowalk M.P., Pavlik V.N., Brown A.E., Zhang S., Raviotta J.M., Moehling K.K., Hawk M., Ricci E.M., Middleton D.B. (2016). Using the 4 pillars™ practice transformation program to increase adult influenza vaccination and reduce missed opportunities in a randomized cluster trial. BMC Infect. Dis..

[4] ECDC Vaccine Schedules in All Countries in the EU/EEA. https://vaccine-schedule.ecdc.europa.eu/.

[5] National Foundation for Infectious Diseases Call to Action: Strategies to Improve Adult Immunization in the US. https://www.nfid.org/resource/call-to-action-strategies-to-improve-adult-immunization-in-the-us/.

[6] World Health Organization The Burden of Influenza. https://www.who.int/news-room/feature-stories/detail/the-burden-of-influenza#:~:text=Nevertheless%2C%20WHO%20estimates%20that%20there,650%20000%20respiratory%20deaths%20annually.&text=On%20the%20other%20hand%2C%20pandemic,from%20another%20animal%20and%20spreads.

[7] World Health Organization COVID-19 Epidemiological Update–15 March 2024. https://www.who.int/publications/m/item/covid-19-epidemiological-update-15-march-2024.

[8] Lafond K.E., Porter R.M., Whaley M.J., Suizan Z., Ran Z., Aleem M.A., Thapa B., Sar B., Proschle V.S., Peng Z. (2021). Global burden of influenza-associated lower respiratory tract infections and hospitalizations among adults: A systematic review and meta-analysis. PLoS Med..

[9] World Health Organization Number of COVID-19 Cases Reported to WHO (Cumulative Total). https://data.who.int/dashboards/covid19/cases.

[10] World Health Organization Number of COVID-19 Deaths Reported to WHO (Cumulative Total). https://data.who.int/dashboards/covid19/deaths.

[11] Janssen C., Mosnier A., Gavazzi G., Combadière B., Crépey P., Gaillat J., Launay O., Botelho-Nevers E. (2022). Coadministration of seasonal influenza and COVID-19 vaccines: A systematic review of clinical studies. Hum. Vaccin. Immunother..

[12] Achterbergh R.C.A., McGovern I., Haag M. (2024). Co-administration of influenza and COVID-19 vaccines: Policy review and vaccination coverage trends in the European Union, UK, US, and Canada between 2019 and 2023. Vaccines.

[13] World Health Organization (2021). Coadministration of Seasonal Inactivated Influenza and COVID-19 Vaccines: Interim Guidance, 21 October 2021.

[14] Higgins J.P.T., Thomas J., Chandler J., Cumpston M., Li T., Page M.J., Welch V.A. (2019). Cochrane Handbook for Systematic Reviews of Interventions.

[15] Page M.J., McKenzie J.E., Bossuyt P.M., Boutron I., Hoffmann T.C., Mulrow C.D., Shamseer L., Tetzlaff J.M., Akl E.A., Brennan S.E. (2021). The PRISMA 2020 statement: An updated guideline for reporting systematic reviews. Int. J. Surg..

[16] Higgins J.P., Altman D.G., Gotzsche P.C., Juni P., Moher D., Oxman A.D., Savovic J., Schulz K.F., Weeks L., Sterne J.A. (2011). The Cochrane Collaboration’s tool for assessing risk of bias in randomised trials. BMJ.

[17] Stang A. (2010). Critical evaluation of the Newcastle-Ottawa scale for the assessment of the quality of nonrandomized studies in meta-analyses. Eur. J. Epidemiol..

[18] Aydillo T., Balsera-Manzanero M., Rojo-Fernandez A., Escalera A., Salamanca-Rivera C., Pachon J., Del Mar Munoz-Garcia M., Sanchez-Cordero M.J., Sanchez-Cespedes J., Garcia-Sastre A. (2024). Concomitant administration of seasonal influenza and COVID-19 mRNA vaccines. Emerg. Microbes Infect..

[19] Baj A., Gasperina D.D., Focosi D., Forlani G., Ferrante F.D., Novazzi F., Azzi L., Maggi F. (2022). Safety and immunogenicity of synchronous COVID19 and influenza vaccination. J. Clin. Virol. Plus.

[20] Gonen T., Barda N., Asraf K., Joseph G., Weiss-Ottolenghi Y., Doolman R., Kreiss Y., Lustig Y., Regev-Yochay G. (2023). Immunogenicity and reactogenicity of coadministration of COVID-19 and influenza vaccines. JAMA Netw. Open.

[21] Harris D.A., Chachlani P., Hayes K.N., McCarthy E.P., Wen K.J., Deng Y., Zullo A.R., Djibo D.A., McMahill-Walraven C.N., Smith-Ray R.L. (2024). COVID-19 and influenza vaccine coadministration among older U.S. adults. Am. J. Prev. Med..

[22] Hause A.M., Zhang B., Yue X., Marquez P., Myers T.R., Parker C., Gee J., Su J., Shimabukuro T.T., Shay D.K. (2022). Reactogenicity of simultaneous COVID-19 mRNA booster and influenza vaccination in the US. JAMA Netw. Open.

[23] Kenigsberg T.A., Goddard K., Hanson K.E., Lewis N., Klein N., Irving S.A., Naleway A.L., Crane B., Kauffman T.L., Xu S. (2023). Simultaneous administration of mRNA COVID-19 bivalent booster and influenza vaccines. Vaccine.

[24] Kim A.S., Kim S.M., Song J.E., Hwang S., Nam E., Kwon K.T. (2023). Adverse reactions after BNT162b2 messenger RNA vaccination for Coronavirus Disease 2019 in healthcare workers compared with influenza vaccination. Vaccines.

[25] Lu Y., Matuska K., Nadimpalli G., Ma Y., Duma N., Zhang H.T., Chiang Y., Lyu H., Chillarige Y., Kelman J.A. (2024). Stroke risk after COVID-19 bivalent vaccination among US older adults. JAMA.

[26] McElvaney O.J., Cleary B., Fraughen D.D., Kelly G., McElvaney O.F., Murphy M.P., Branagan P., Gunaratnam C., Carroll T.P., Goss C.H. (2024). Safety and reactogenicity of COVID-19 vaccination in severe alpha-1 antitrypsin deficiency. Chronic Obstr. Pulm. Dis..

[27] McGrath L.J., Malhotra D., Miles A.C., Welch V.L., Di Fusco M., Surinach A., Barthel A., Alfred T., Jodar L., McLaughlin J.M. (2023). Estimated effectiveness of coadministration of the BNT162b2 BA.4/5 COVID-19 vaccine with influenza vaccine. JAMA Netw. Open.

[28] Moro P.L., Zhang B., Ennulat C., Harris M., McVey R., Woody G., Marquez P., McNeil M.M., Su J.R. (2023). Safety of co-administration of mRNA COVID-19 and seasonal inactivated influenza vaccines in the vaccine adverse event reporting system (VAERS) during July 1, 2021–June 30, 2022. Vaccine.

[29] Moscara L., Venerito V., Martinelli A., Di Lorenzo A., Toro F., Violante F., Tafuri S., Stefanizzi P. (2023). Safety profile and SARS-CoV-2 breakthrough infections among HCWs receiving anti-SARS-CoV-2 and influenza vaccines simultaneously: An Italian observational study. Vaccine.

[30] Pascucci D., Lontano A., Regazzi L., Marziali E., Nurchis M.C., Raponi M., Vetrugno G., Moscato U., Cadeddu C., Laurenti P. (2023). Co-administration of SARS-CoV-2 and influenza vaccines in healthcare workers: Results of two vaccination campaigns in a large teaching hospital in Rome. Human. Vaccines Immunother..

[31] Radner H., Sieghart D., Jorda A., Fedrizzi C., Hasenohrl T., Zdravkovic A., Redlberger-Fritz M., Puchammer-Stoeckl E., Anderle K., Bergmann F. (2023). Reduced immunogenicity of BNT162b2 booster vaccination in combination with a tetravalent influenza vaccination: Results of a prospective cohort study in 838 health workers. Clin. Microbiol. Infect..

[32] Venuto R., Giunta I., Cortese R., Denaro F., Panto G., Privitera A., D’Amato S., Genovese C., La Fauci V., Fedele F. (2022). The importance of COVID-19/influenza vaccines co-administration: An essential public health tool. Infect. Dis. Rep..

[33] Dulfer E.A., Geckin B., Taks E.J.M., GeurtsvanKessel C.H., Dijkstra H., van Emst L., van der Gaast-de Jongh C.E., van Mourik D., Koopmans P.C., Domínguez-Andrés J. (2023). Timing and sequence of vaccination against COVID-19 and influenza (TACTIC): A single-blind, placebo-controlled randomized clinical trial. Lancet Reg. Health Eur..

[34] Lazarus R., Baos S., Cappel-Porter H., Carson-Stevens A., Clout M., Culliford L., Emmett S.R., Garstang J., Gbadamoshi L., Hallis B. (2021). Safety and immunogenicity of concomitant administration of COVID-19 vaccines (ChAdOx1 or BNT162b2) with seasonal influenza vaccines in adults in the UK (ComFluCOV): A multicentre, randomised, controlled, phase 4 trial. Lancet.

[35] Murdoch L., Quan K., Baber J.A., Ho A.W.Y., Zhang Y., Xu X., Lu C., Cooper D., Koury K., Lockhart S.P. (2023). Safety and immunogenicity of the BNT162b2 vaccine coadministered with seasonal inactivated influenza vaccine in adults. Infect. Dis. Ther..

[36] Ramsay J.A., Jones M., Vande More A.M., Hunt S.L., Williams P.C.M., Messer M., Wood N., Macartney K., Lee F.J., Britton W.J. (2023). A single blinded, phase IV, adaptive randomised control trial to evaluate the safety of coadministration of seasonal influenza and COVID-19 vaccines (The FluVID study). Vaccine.

[37] Choi M.J., Yu Y.J., Kim J.W., Ju H.J., Shin S.Y., Yang Y.J., Cheong H.J., Kim W.J., Kim C., Kim H.J. (2024). Immunogenicity and safety of concomitant bivalent COVID-19 and quadrivalent influenza vaccination: Implications of immune imprinting and interference. Clin. Microbiol. Infect..

[38] Beccia F., Lontano A., Rossi M.F., Marziali E., Pascucci D., Raponi M., Santoro P.E., Moscato U., Laurenti P. (2023). Three-year COVID-19 and flu vaccinations among medical residents in a tertiary hospital in Italy: The threat of acceptance decline in seasonal campaigns. Human. Vaccines Immunother..

[39] Kim A.S., Kim S.M., Song J., Hwang S., Nam E., Kwon K.T. (2022). Adverse reactions following third dose of the BNT162b2 mRNA COVID-19 vaccine compared with influenza vaccine in healthcare workers. Infect. Chemother..

[40] AstraZeneca FluMist (Influenza Vaccine Live, Intranasal). https://www.flumist.com/.

[41] Moss S., Jurkowicz M., Nemet I., Atari N., Kliker L., Abd-Elkader B., Gonen T., Martin E.T., Lustig Y., Regev-Yochay G. (2023). Immunogenicity of co-administered omicron BA.4/BA.5 bivalent COVID-19 and quadrivalent seasonal influenza vaccines in Israel during the 2022–2023 winter season. Vaccines.

[42] World Health Organization Interim recommendations on COVID-19 vaccination in autumn 2022 for the WHO European Region: Conclusions and recommendations of the European Technical Advisory Group of Experts on Immunization: Ad hoc virtual meeting. Proceedings of the Interim Recommendations on COVID-19 Vaccination in Autumn 2022 for the WHO European Region: Conclusions and Recommendations of the European Technical Advisory Group of Experts on Immunization: Ad hoc Virtual Meeting.

[43] Gianfredi V., Pennisi F., Lume A., Ricciardi G.E., Minerva M., Riccò M., Odone A., Signorelli C. (2021). Challenges and opportunities of mass vaccination centers in COVID-19 times: A rapid review of literature. Vaccines.

[44] Goralnick E., Kaufmann C., Gawande A.A. (2021). Mass-vaccination sites—An essential innovation to curb the COVID-19 pandemic. N. Engl. J. Med..

[45] Haviari S., Bénet T., Saadatian-Elahi M., André P., Loulergue P., Vanhems P. (2015). Vaccination of healthcare workers: A review. Hum. Vaccines Immunother..

[46] Okpani A.I., Lockhart K., Barker S., Grant J.M., Yassi A. (2024). Did the health care vaccine mandate work? An evaluation of the impact of the COVID-19 vaccine mandate on vaccine uptake and infection risk in a large cohort of Canadian health care workers. Am. J. Infect. Control.

[47] World Health Organization (2022). Implementation Guide for Vaccination of Health Workers.

[48] Plotkin S.A. (2010). Correlates of protection induced by vaccination. Clin. Vaccine Immunol..

[49] Al-Worafi Y.M. (2023). Healthcare facilities in developing countries: Infrastructure. Handbook of Medical and Health Sciences in Developing Countries: Education, Practice, and Research.

