# Correction: Boikos et al. Co-Administration of BNT162b2 COVID-19 and Influenza Vaccines in Adults: A Global Systematic Review. *Vaccines* 2025, *13*, 381

**DOI:** 10.3390/vaccines14030198

**Published:** 2026-02-24

**Authors:** Constantina Boikos, Kassandra Schaible, Solange Nunez-Gonzalez, Verna Welch, Tianyan Hu, Moe Hein Kyaw, Laura Elizabeth Choi, Joanna Kamar, Henry Goebe, John McLaughlin

**Affiliations:** 1 Pfizer Inc., Kirkland, QC H9J 2M5, Canada; 2 Evidera, Thermo Fisher Scientific, Waltham, MA 02451, USA; kassandra.schaible@thermofisher.com (K.S.); solange.gonzalez@thermofisher.com (S.N.-G.); joanna.kamar@thermofisher.com (J.K.); 3 Pfizer Inc., New York, NY 10001, USA; verna.welch@pfizer.com (V.W.); tianyan.hu@pfizer.com (T.H.); moe.kyaw@pfizer.com (M.H.K.); 4 Pfizer Inc., Collegeville, PA 19426, USA; laura.choi@pfizer.com (L.E.C.); john.mclaughlin.phd@gmail.com (J.M.); 5 Pfizer Inc., Tadworth KT20 7NS, UK; henry.goebe@pfizer.com

In the original publication [1], there was a mistake in Figure 4. The data presented in the Figure did not correspond to the data as presented in Appendix A, which impacted the ranges presented in the associated text in Section 3.5 “Safety/Reactogenicity of Same-day Co-administration”, paragraphs 2–4. The text, Figure 4, and cited references have been corrected below. 

The 2nd sentence of the 2nd paragraph in Section 3.5, “…and BNT162b2 booster alone ranged from 49% [20] to 52% [36]”, should be corrected to “and BNT162b2 booster alone ranged from 49% [20] to 64% [22]”.

The 2nd sentence of the 3rd paragraph in Section 3.5, “…BNT162b2 booster followed by SIV later ranged from 33% [26] to 94% [34], and BNT162b2 booster alone ranged from 27% [20] to 82% [26]”, should be corrected to “BNT162b2 booster followed by SIV later ranged from 50% [26] to 82% [34], and BNT162b2 booster alone ranged from 27% [20] to 58.9% [22]”.

The 2nd sentence of the 4th paragraph in Section 3.5, “…BNT162b2 booster followed by SIV later ranged from 50% [26] to 82% [34], and BNT162b2 booster alone ranged from 47% [20] to 97.7% [26]” should be corrected to “…SIV followed by BNT162b2 booster later ranged from 29% [35] to 56% [29] and BNT162b2 booster alone ranged from 71% [19] to 97.7% [31]”.

**Figure 4:**
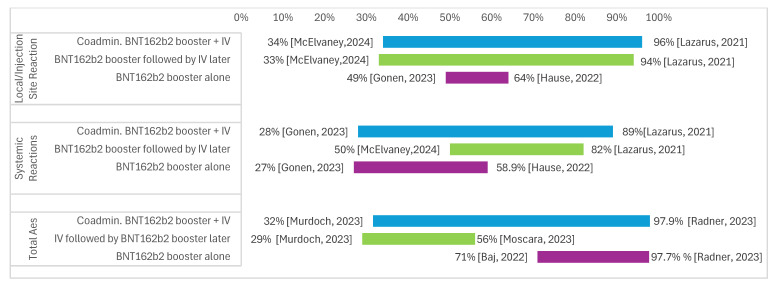
Safety/reactogenicity outcome ranges. Abbreviations: Ae = adverse event, IV = influenza vaccine [19,20,22,26,29,31,34,35].

The authors state that the scientific conclusions are unaffected. This correction was approved by the Academic Editor. The original publication has also been updated.
